# Reduced Graphene Oxides Modulate the Expression of Cell Receptors and Voltage-Dependent Ion Channel Genes of Glioblastoma Multiforme

**DOI:** 10.3390/ijms22020515

**Published:** 2021-01-06

**Authors:** Jaroslaw Szczepaniak, Joanna Jagiello, Mateusz Wierzbicki, Dorota Nowak, Anna Sobczyk-Guzenda, Malwina Sosnowska, Slawomir Jaworski, Karolina Daniluk, Maciej Szmidt, Olga Witkowska-Pilaszewicz, Barbara Strojny-Cieslak, Marta Grodzik

**Affiliations:** 1 Department of Nanobiotechnology, Institute of Biology, Warsaw University of Life Sciences, 02-787 Warsaw, Poland; jaroslaw_szczepaniak@sggw.edu.pl (J.S.); mateusz_wierzbicki@sggw.edu.pl (M.W.); malwina_sosnowska@sggw.edu.pl (M.S.); slawomir_jaworski@sggw.edu.pl (S.J.); karolina_daniluk@sggw.edu.pl (K.D.); 2 Department of Chemical Synthesis and Flake Graphene, Łukasiewicz Research Network–Institute of Microelectronics and Photonics, 01-919 Warsaw, Poland; joanna.jagiello@itme.edu.pl; 3 Tricomed SA, 5/9 Swientojanska St., 93-493 Lodz, Poland; nowak.dora@gmail.com; 4 Institute of Materials Science and Engineering, Lodz University of Technology, 90-924 Lodz, Poland; anna.sobczyk-guzenda@p.lodz.pl; 5 Department of Morphologic Sciences, Institute of Veterinary Medicine, Warsaw University of Life Sciences, 02-787 Warsaw, Poland; maciej_szmidt@sggw.edu.pl; 6 Department of Pathology and Veterinary Diagnostics, Institute of Veterinary Medicine, Warsaw University of Life Sciences, 02-787 Warsaw, Poland; olga_witkowska_pilaszewicz@sggw.edu.pl

**Keywords:** graphene, reduced graphene oxide, glioblastoma multiforme, voltage-gated ion channel, cell membrane receptor, membrane potential

## Abstract

The development of nanotechnology based on graphene and its derivatives has aroused great scientific interest because of their unusual properties. Graphene (GN) and its derivatives, such as reduced graphene oxide (rGO), exhibit antitumor effects on glioblastoma multiforme (GBM) cells in vitro. The antitumor activity of rGO with different contents of oxygen-containing functional groups and GN was compared. Using FTIR (fourier transform infrared) analysis, the content of individual functional groups (GN/exfoliation (ExF), rGO/thermal (Term), rGO/ammonium thiosulphate (ATS), and rGO/ thiourea dioxide (TUD)) was determined. Cell membrane damage, as well as changes in the cell membrane potential, was analyzed. Additionally, the gene expression of voltage-dependent ion channels (*clcn3*, *clcn6*, *cacna1b*, *cacna1d*, *nalcn*, *kcne4*, *kcnj10*, and *kcnb1*) and extracellular receptors was determined. A reduction in the potential of the U87 glioma cell membrane was observed after treatment with rGO/ATS and rGO/TUD flakes. Moreover, it was also demonstrated that major changes in the expression of voltage-dependent ion channel genes were observed in *clcn3*, *nalcn*, and *kcne4* after treatment with rGO/ATS and rGO/TUD flakes. Furthermore, the GN/ExF, rGO/ATS, and rGO/TUD flakes significantly reduced the expression of extracellular receptors (uPar, CD105) in U87 glioblastoma cells. In conclusion, the cytotoxic mechanism of rGO flakes may depend on the presence and types of oxygen-containing functional groups, which are more abundant in rGO compared to GN.

## 1. Introduction

Glioblastoma (GBM) is the most common primary malignant brain tumor. GBM is associated with poor prognosis and a life expectancy of approximately 15 months despite optimal therapy, which includes surgery, chemotherapy, and radiotherapy [1]. The lack of therapeutic success has been attributed to a variety of factors, including rapid infiltration of brain tumor cells, inter- and intratumor heterogeneity, limited diffusion of therapeutic drugs across the blood–brain barrier and brain parenchyma/tumor, and the presence of GBM stem cells (GSC) in the tumor, which are resistant to radiotherapy and chemotherapy and are capable of tumor formation and indefinite self-renewal [2].

Graphene is made up of a layer of carbon atoms arranged in a hexagonal pattern and consists purely of sp2 hybridized bonds. It has gained enormous interest in various fields owing to its unique electrochemical properties, which include high thermal conductivity, high current, density, chemical volume, optical transmittance, and very high hydrophobicity [3,4]. It is the simplest form of carbon and the thinnest material ever produced [5]. The graphene family includes sheets and flakes of graphene as well as graphene oxide (GO) and reduced graphene oxide (rGO) [6,7]. GO is highly hydrophilic because of the presence of a large number of oxygen groups on the surface (hydroxyl, carboxyl, epoxy). rGO has more oxygen functional groups than graphene (GN), but less than GO. Therefore, rGO is less hydrophilic than GO and, therefore, has higher electrical conductivity [8]. In addition to the numerous applications of graphene materials in electronics, their derivatives are also assessed for their use in medicine, for example, in anticancer therapy [9,10]. Our previous studies have shown that graphene and its derivatives can be cytotoxic to glioblastoma cells in vitro and in vivo. GN, rGO, and GO induce apoptosis and lead to the reduction of viability and proliferation in U87 and U118 glioblastoma cell lines [11]. Jaworski et al. also showed that graphene flakes were too large (0.45–1.5 µm) to enter the glioblastoma cells [11,12]. However, both GN and rGO can activate the mitochondrial-dependent apoptotic pathway by reducing the mitochondrial membrane potential in U87 glioma cells. On the contrary, GO can regulate the expression of mitochondrial oxidative phosphorylation (OXPHOS) genes in GBM, thus leading to a decrease in the invasion potential of cancer cells [13]. The tested materials (GN/exfoliation (ExF), rGO/thermal (Term), rGO/ammonium thiosulphate (ATS), and rGO/ thiourea dioxide (TUD)) showed no effect on the cell cycle [14]. Despite the fact that even minimal oxygen content on the surface of the flakes may reduce the proapoptotic abilities of graphene and its derivatives, oxygen-containing derivatives have a better affinity for GBM cells than pure graphene, which allows for better targeting of the intended effect [15].

An important finding seems to be the reduction of oxygen content in rGO in relation to the starting material, GO, which results in an increase of delocalized electrons on the surface of the graphene flakes. This can lead to a disruption of the signaling pathways in the plasma membrane or to direct interaction with cell structures, which are sensitive to electrochemical potential. Therefore, graphene and its derivatives are characterized by strong bioelectric properties; because of the presence of delocalized electrons and oxygen-containing functional groups on the surface of the flakes, this material can interact with structures that receive electrical signals, for example, receptors located on the cell surface, as well as proteins building voltage-dependent ion channels, ultimately causing changes in the potential of the cell membrane. Cell surface charge is a key biophysical parameter that depends on the composition of the cytoplasmic membrane and the physiological state of cells. In addition to the presence of ion channels and transporters, negative values of cell membrane potential at physiological pH values are also caused by the presence of nonionic groups in phospholipids (phosphatidylcholine; −62 mV), proteins, and their polysaccharide conjugates [16]. Direct comparisons of the in vitro and in vivo Vm levels of normal and cancer cells showed that the cancer cells were more depolarized (negative Vm) than their normal counterparts [17]. Therefore, the studied materials of graphene origin (GN and rGO) may prefer adhesion to neoplastic cells with depolarized cell membranes. Moreover, Fiorillo et al. proved that GO, characterized by the presence of many functional groups containing oxygen, preferred adhesion to U87 tumor cells rather than stem cells and normal fibroblasts [18]. Therefore, it has been hypothesized that graphene-derived flakes exert modulating and transducing effects on proteins contained in the membrane of U87 glioma cancer cells. These effects result from the unique structure and type of functional groups on the surface of graphene. GN flakes are characterized by several functional groups on the surface, and they possess a larger electron cloud than rGO.

Ion channels play an important role in the regulation of electrical excitability in normal and cancer cells [19]. Ion channels and transporters are also associated with GBM tumor growth and malignancy [20]. Genomic analysis revealed that the genes involved in Na, K, Ca transmission or transport belong to the most frequently mutated functional groups affecting GBM in 90% of the tested samples [21,22]. Therefore, the article discusses the subject of studying the expression of individual subunits that build ion channels (Na^+^, K^+^, Ca^2+^, Cl^−^). The functioning of ion channels and pumps influences the migration and proliferation of GBM cells. For example, deregulated K^+^ and Cl^−^ channels regulate the osmotic drive, allowing for cell shape and volume changes that promote glioblastoma cell migration [23], and Ca-activated K^+^ (BK) channels, which control glioblastoma cell growth [24]. In this study, we hypothesize that flakes of graphene (GN) and reduced graphene oxides (rGOs) may affect the expression of voltage-dependent ion channel genes Cl^−^ (*clcn3* and *clcn6*), Ca^2+^ (*cacna1b* and *cacna1d*), Na^+^ (*nalcn*), K^+^ (*kcne4*, *kcnj10*, and *kcnb1*), resulting in an alteration of the potential of the glioblastoma cell membrane. It is also suspected that GN and rGO flakes may reduce the expression of extracellular receptors and further reduce the invasiveness of glioblastoma in vitro.

The aim of this study is to determine changes in the cell membrane caused by direct or indirect contact with bioelectric flakes of rGO with different degrees of reduction compared to GN in U87 glioblastoma multiforme cells in vitro.

## 2. Results

### 2.1. Physicochemical Characterization of GN and rGO Flakes

The physicochemical properties (inter alia, transmission electron microscopy, Raman spectroscopy, atomic force microscopy, zeta potential, oxygen content, and electrical resistance) of graphene (GN) and reduced graphene oxides (rGOs) have recently been published [14]. In this study, the physicochemical properties were complemented with an additional analysis of the tested materials. FTIR analysis allowed us to obtain detailed information about the functional groups.

**Figure 1:**
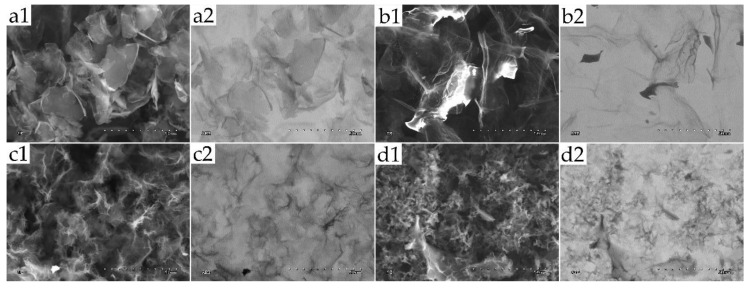
Morphology of GN and rGOs was examined using a scanning electron microscope (SEM). (**a1**) GN/ExF, (**b1**) rGO/Term, (**c1**) rGO/ATS, (**d1**) rGO/TUD—standard SEM images show 2D nanosheets morphologies. Scale bare: 50 µm. (**a2**) GN/ExF, (**b2**) rGO/Term, (**c2**) rGO/ATS, (**d2**) rGO/TUD—AEE mode showing conductive materials. Scale bare: 50 µm. **Abbreviations**: GN, graphene; rGO, reduced graphene oxide; SEM, scanning electron microscope; AEE, active emission element; ExF, exfoliation; Term, thermal; ATS, ammonium thiosulphate; TUD, thiourea dioxide.

SEM images were used to confirm the morphology of the graphene flakes. Microphotographs of thin layers of GN and rGO graphene sheets are shown in Figure 1. In GN/ExF (a1), irregular edges of graphene flakes were observed. Photos showing rGO/Term (b1), rGO/ATS (c1), and rGO/TUD (d1) samples showed wrinkled and complex textures with rough surfaces, resulting from collapse. SEM images, taken in the AEE (active emission element) mode (Figure 1(a2,b2,c2,d2)), allowed the scanning of graphene with the table current. Owing to this, the quality of the images was good for conductive materials.

**Figure 2:**
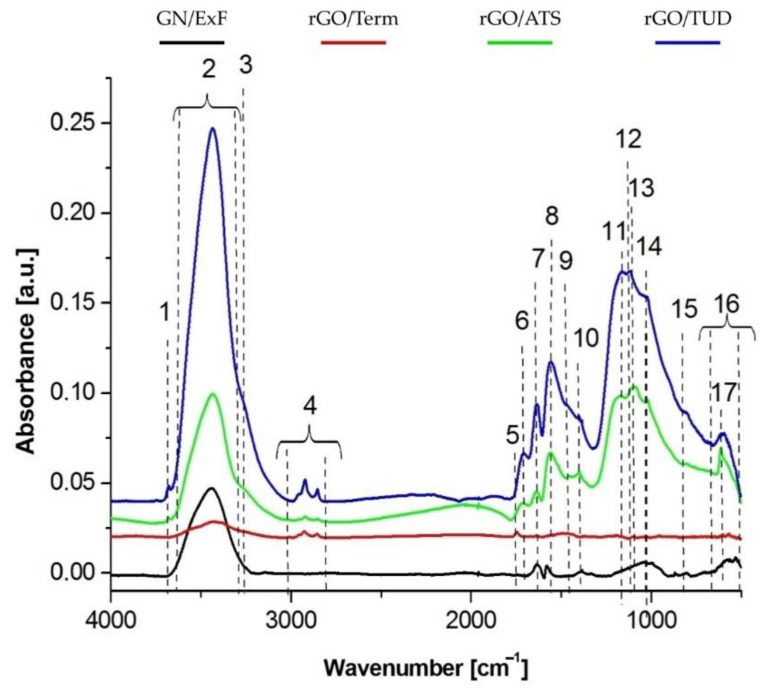
Fourier transform infrared (FTIR) spectra of graphene (GN/ExF; black line) and reduced graphene oxides (rGO/Term, red line; rGO/ATS, green line; rGO/TUD, blue line). The individual digital markings on the graph show the bands associated with the occurrence of individual functional groups; 1 (OH-), 2 (OH-), 3 (N-H), 4 (C-H), 5 (C=O), 6 (C=O), 7 (C=C), 8 (COO-), 9 (C-H), 10 (-OH), 11 (C-O-C), 12 (C-NH_2_/C-N), 13 (C=S), 14 (carbon ring), 15 (CH_2_=CH), 16 (carbon ring), 17 (C-S/S-O). **Abbreviations**: GN, graphene; rGO, reduced graphene oxide; FTIR, Fourier transform infrared; a.u., absorbance unit; ExF, exfoliation; Term, thermal; ATS, ammonium thiosulphate; TUD, thiourea dioxide.

The presence of functional groups was confirmed by FTIR analysis (Figure 2). Spectrum analysis showed the presence of aliphatic groups containing C-H bonds in their chemical structure. From the presented spectra, it can be concluded that the purest was graphene GN/ExF. The spectrum of this sample lacked absorption bands in the range of wave numbers between 3000–2800 cm^−1^ and 1470–1440 cm^−1^. Graphene rGO/Term and rGO/ATS had similar amounts of these impurities. The most C-H bonds occurring in both the form of methyl and methylene groups were in graphene rGO/TUD. In the obtained spectra, bands originating mainly from O-H, C-H, C=O, and C-O bond vibrations were visible. The location and assignment of individual bands to specific bonds are shown and summarized in Table 1.

Considering the quantity of oxidized carbon in its chemical structure as the criterion for assessing the quality of the tested material, the largest number of such connections was found in graphene rGO/TUD. Slightly less connection was observed in graphene rGO/ATS. Graphene GN/ExF had two characteristic low-intensity peaks belonging to C=O and COO- bonds, which were also present in the spectra of samples rGO/ATS and rGO/TUD. The least number of C=O bonds occurred in the graphene rGO/Term. Only one peak with a maximum at 1747 cm^−1^ appeared in the spectrum of this material. Therefore, the rGO/Term material turned out to be the most hydrophobic, which was confirmed by macroscopic observation of the behavior of graphene flakes in this solution. The sharp maximum in graphene rGO/TUD was observed at 3680 cm^−1^. Additionally, bands from the vibration of N-H and C-NH_2_ bonds appear in the spectra of graphene rGO/ATS and rGO/TUD, which were found at 3280 and 1124 cm^−1^, respectively. The sulfur component was also incorporated into the graphene structure, giving peaks at wave numbers 1100 and 617 cm^−1^.

### 2.2. Membrane Potential

**Figure 3:**
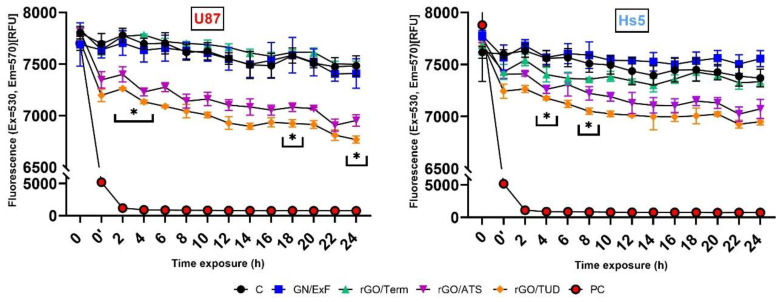
Effect of graphene flakes (GN/ExF) and three types of reduced graphene oxide flakes (rGO/Term, rGO/ATS, and rGO/TUD) on the membrane potential of U87 and Hs5 cell lines. The immediate reading after the addition is marked as 0 on the *x*-axis of the membrane potential sensor. PC represents the group after H_2_O_2_ (hydrogen peroxide) treatment as positive control. The reading after 30 min incubation with a membrane potential sensor is marked as 0′ on the *x*-axis. The readings were taken for 24 h at 2 h intervals. Values are expressed as mean ± standard deviation. Statistical significance between the control and treated cells is indicated by an asterisk and was assessed using Bonferroni’s multiple comparisons test. Differences with *p*-value < 0.05 were considered significant. One asterisk (*) means *p*-value < 0.05. **Abbreviations**: rGO, reduced graphene oxide; GN, graphene; C, control group (untreated group); PC, positive control; h, hours; ExF, exfoliation; Term, thermal; ATS, ammonium thiosulphate; TUD, thiourea dioxide.

In the next stage, changes in cell membrane potential were analyzed. The results are presented in the form of line graphs depicting changes in cell membrane potential (Figure 3). In glioblastoma U87 cells, a statistically significant decrease in cell membrane potential was observed only after treatment with rGO/ATS and rGO/TUD flakes. The first potential peak (decrease; 516.7-563 RFU (relative fluorescence units)) after rGO/TUD treatment was observed after 2 and 4 h (*p* < 0.05). Maximum potential reduction after rGO/TUD treatment (714.7 RFU) occurred after 24 h of treatment (*p* < 0.05). In U87 cells after rGO/ATS treatment (567 RFU), the maximum reduction in cell membrane potential was observed after 22 h. The strongest reduction was observed after 18 h for both rGO/ATS and rGO/TUD (*p* < 0.05). In Hs5 cells, a significant reduction in cell membrane potential was observed only for rGO/ATS flakes (maximum 297.7 RFU after 4 h of treatment; *p*-value <0.05). The strongest reduction was observed after 8 h of rGO/ATS flake treatment (*p* < 0.05). Greater changes in the potential were observed in cancer cells (421 RFU, 561.7 RFU) than in healthy cells (258.4 RFU, 384.3 RFU) after rGO/ATS and rGO/TUD treatment.

### 2.3. Gene Expression of Voltage-Gated Ion Channels

**Figure 4:**
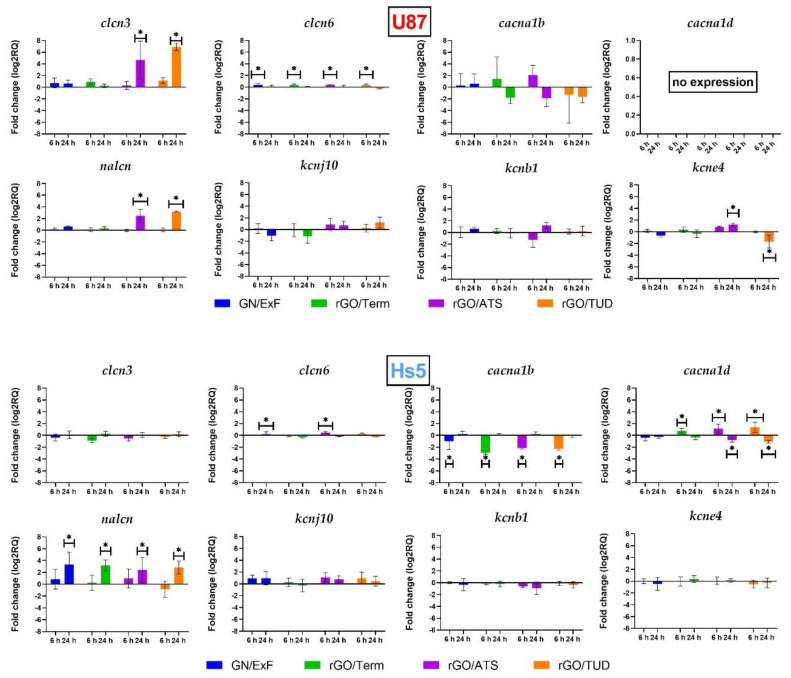
Analysis of the mRNA expression level of voltage-gated ion channels after treatment at 25 µg/mL concentration for 6 and 24 h in U87 and Hs5 cells. The results are calculated relative to the control values. Log2RQ (log2 relative quantitation) values for all genes are normalized to the RPL13A housekeeping gene. Statistical significance between the control and the treated cells is indicated by an asterisk and was assessed using Bonferroni’s multiple comparisons test. Differences with *p*-value < 0.05 were considered significant. One asterisk (*) means *p*-value < 0.05. **Abbreviations**: rGO, reduced graphene oxide; GN, graphene; C, control group (untreated group); ExF, exfoliation; Term, thermal; ATS, ammonium thiosulphate; TUD, thiourea dioxide; *clcn3*, chloride voltage-gated channel 3; *clcn6*, chloride voltage-gated channel 6; *cacna1b*, calcium voltage-gated channel subunit alpha1 B; *cacna1d*, calcium voltage-gated channel subunit alpha1 D; *nalcn*, sodium leak channel, non-selective; *kcnj10*, potassium inwardly rectifying channel subfamily J member 10; *kcnb1*, potassium voltage-gated Channel subfamily B member 1; *kcne4*, potassium voltage-gated channel subfamily E regulatory subunit 4; rpl13a, ribosomal protein L13a; log2RQ, log2 relative quantitation.

Analysis of the expression of genes involved in the transport of chlorine, calcium, sodium, and potassium voltage-dependent ions was performed. The results are shown in Figure 4 and Table S1. Gene expression that was studied using real-time PCR was focused on the genes coding the individual subunits of proteins that cocreate ion channels, responsible for the transport of the aforementioned ions.

#### 2.3.1. Chloride Channels

The expression of two subunits of chloride channels, *clcn3* and *clcn6*, was measured. In the U87 line, there was an increase in *clcn3* and *clcn6* expression 24 h after rGO/ATS (log2RQ = 4.67 +/− 0.7158) and rGO/TUD (log2RQ = 6.89 +/− 0.7158) treatment. There was an increase in *clcn3* expression after 6 h of treatment in all groups. The highest increase was observed in the rGO/ATS group (log2RQ = 0.42 +/− 0.1063). In Hs5 cells, there was no increase in *clcn3* and *clcn6* expression after 24 h treatment; however, there was higher expression after 6 h of treatment with rGO/ATS flakes (log2RQ = 0.42 +/− 0 1063).

#### 2.3.2. Calcium Channels

In the noncancerous Hs5 cell line, there was an increase in *cacna1d* expression after 6 h of graphene rGO/Term treatment (log2RQ = 0.7957 +/− 0.2863), rGO/ATS (log2RQ = 1.160 +/− 0.2863), and rGO/TUD (log2RQ = 1.387 +/− 0.2863). After 24 h, there was a decrease in *cacna1d* expression in the rGO/ATS (log2RQ = −0.7671 +/− 0.2863) and rGO/TUD (log2RQ = −1.029 +/− 0.2863) groups. There was no expression of the *cacna1d* gene in the U87 glioblastoma line even in the control sample.

Comparing the expression of the *cacna1b* gene in both cell lines, a much higher expression was observed in the noncancerous Hs5 cells (log2RQ = 7.757 +/− 1.492). Additionally, in this cell line, under the influence of graphene flakes, a significant reduction in *cacna1b* expression was observed in all groups after 6 h of treatment. The highest reduction occurred in the rGO/Term treatment (log2RQ = −2.953 +/− 0.2990). After 24 h of treatment, no statistically significant changes were observed.

#### 2.3.3. Sodium Channels

In the Hs5 cell line, there was an increase in *nalcn* expression in all examined groups after 24 h of flake treatment. The expression changes were as follows: GN/ExF (log2RQ = 3.810 +/− 0.978), rGO/Term (log2RQ = 3.683 +/− 0.978), rGO/ATS (log2RQ = 2.904 +/− 0.978), and rGO/TUD (log2RQ = 3.338 +/− 0.978). After 6 h of treatment, no significant changes were observed. In the U87 cell line, an increase in *nalcn* expression was observed after 24 h of treatment with rGO/ATS (log2RQ = 2.472 +/− 0.978) and rGO/TUD (log2RQ = 3.200 +/− 0.978) flakes. There were no significant changes in *nalcn* gene expression after 6 h of treatment in the U87 cell line.

#### 2.3.4. Potassium Channels

In our study, we analyzed the expression of two potassium ion channel genes (*kcnb1* and *kcne4*). For both Hs5 fibroblast cells and U87 tumor cells, no statistically significant changes in *kcnb1* gene expression were observed. In Hs5 cells, there was no statistically significant change in *kcne4* expression in any of the studied groups. In U87, there was an increase in *kcne4* expression after 24 h treatment by rGO/ATS flakes (log2RQ = 1.192 +/− 0.33452) and a decrease (log2RQ = −1.734 +/− 0.33452) after 24 h of rGO/TUD treatment. No statistically significant changes in the expression of the *kcnj10* gene were observed in either Hs5 fibroblast cells or U87 tumor cells.

### 2.4. Expression of Membrane Receptors

**Figure 5:**
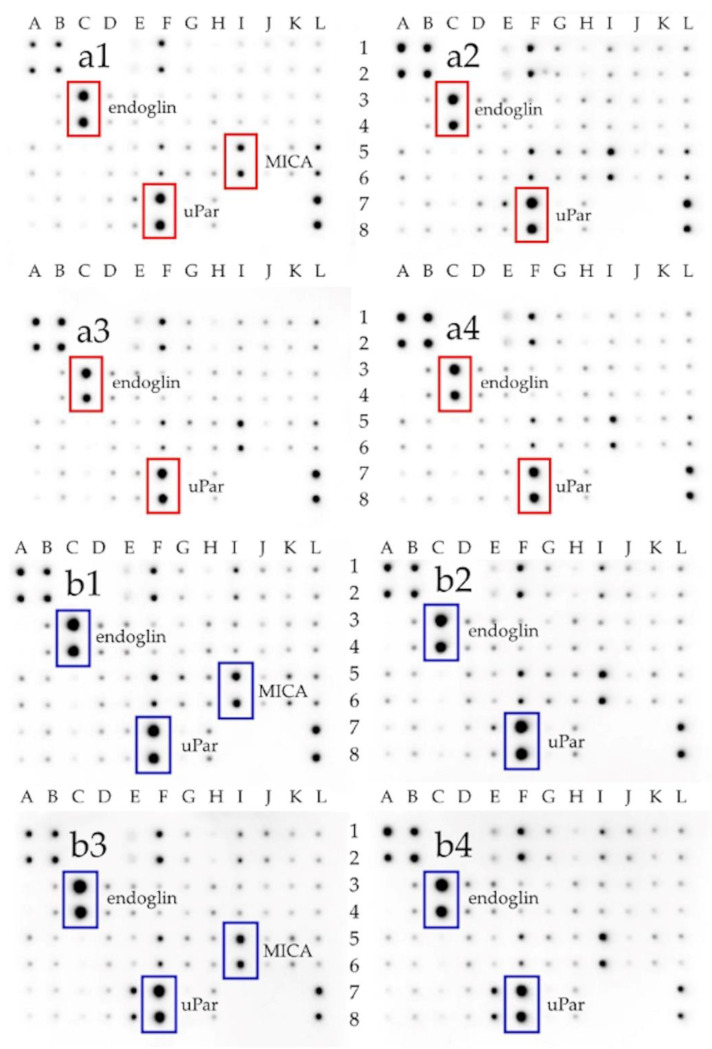
Antibody array analysis of human cell membrane receptor (original drafts) in U87 glioma cells (**a1**–**a4**) and Hs5 fibroblast cells (**b1**–**b4**), with or without 24 h treatment (C: control, **a1**,**b1**), with graphene flakes (GN/ExF: **a2**,**b2**) and two types of reduced graphene oxides (rGO/ATS: **a3**,**b3**; rGO/TUD: **a4**,**b4**). The full array map and uncropped images are available in the supplement material. Results were normalized and compared to a dots control sample. The dots with the location (C3, C4) indicate the expression of endoglin (CD105), (F7, F8) indicate uPar (CD87), and (I5, I6) indicate MICA. **Abbreviations**: rGO, reduced graphene oxide; GN, graphene; C, control group (untreated group); ExF, exfoliation; Term, thermal; ATS, ammonium thiosulphate; TUD, thiourea dioxide; CD105, endoglin; uPar, urokinase plasminogen activator surface receptor; MICA, major histocompatibility complex class I chain-related protein A.

Extracellular receptors expression was analyzed using Human Receptor Antibody Array (Targets: 4-1BB (TNFRSF9), ALCAM (CD166), CD80 (B7-1), BCMA (TNFRSF17), CD14, CD30 (TNFRSF8), CD40 Ligand (TNFSF5), CEACAM-1, DR6 (TNFRSF21), Dtk, Endoglin (CD105), ErbB3, E-Selectin, Fas (TNFRSF6), Flt-3 Ligand, GITR (TNFRSF18), HVEM (TNFRSF14), ICAM-3 (CD50), IL-1 R4 (ST2), IL-1 R1, IL10 Rbeta, IL-17RA, IL-1 R gamma, IL-21R, LIMPII, Lipocalin-2 (NGAL), L-Selectin (CD62L), LYVE-1, MICA, MICB, NRG1-beta 1, PDGF R beta, PECAM-1 (CD31), RAGE, TIM-1 (KIM-1), TRAIL R3 (TNFRSF10C), Trappin-2, uPar, VCAM-1 (CD106), XEDAR) (Table S2). The results are shown in Figure 5 and Figure S1. Significant changes in the expression of endoglin, MICA, and uPar receptors were seen in Hs5 cells. An increase in the expression of the uPar receptor in the Hs5 cell line occurred in all treatment groups. On the contrary, in U87 tumor cells, the effect is the opposite. A reduction in uPar receptor expression in all the studied groups was noticed. In Hs5, there was an increase in CD105 receptor expression in the rGO/ATS and rGO/TUD groups. In U87, there was a decrease in the expression of the CD105 receptor in all groups.

## 3. Discussion

Previous studies have shown that reduced graphene oxides (rGOs), compared to graphene GN, does not exert identical effects on glioblastoma multiforme cancer cells. The effectiveness of the tested materials was examined in terms of viability, metabolic activity, cell cycle dynamics, and the level of apoptosis. The results indicate that GN and rGOs activate the mitochondria-dependent apoptotic pathway by reducing the potential of the mitochondrial membrane. This study has proved that rGOs have a stronger cytotoxic effect than GN. We used the MTT assay to assess whether graphene and its derivatives also affect the viability and metabolic activity of fibroblast Hs5 and glioblastoma U87 cells. The highest decrease in metabolic activity in glioblastoma U87 cells, at 8.69% ± 12.88%, was found in the group treated with rGO/TUD at a concentration of 100 μg/mL. Interestingly, in the group treated with rGO/ATS, the lowest viability, at 37.7% ± 12.55%, occurred at a concentration of 5 μg/mL rGO/ATS. In other groups treated with GN/ExF and rGO/Term, ~50% mortality was observed at concentrations ranging from 25 to 100 μg/mL. Reducing the oxygen content and increasing defects in the connections between carbons in rGOs compared to GO resulted in an increase in the number of delocalized electrons on the surface of the graphene flakes and oxygen groups, including hydroxyl, carboxyl, and epoxy [14]. It was hypothesized that the cytotoxicity of rGOs may mainly result from direct contact with the glioblastoma cell membrane and may lead to the disruption of signaling pathways in the plasma membrane or direct interaction with cell structures, which are sensitive to electrochemical potential (cell membrane, e.g., ion channels and extracellular receptors). Studying the interactions between graphene materials and cell membranes may reveal the underlying mechanisms of the cytotoxicity of these materials.

Using FTIR analysis, a detailed examination was performed to compare the presence of characteristic functional groups in both reduced graphene oxides (rGO/Term, rGO/ATS, rGO/TUD) and graphene (GN/ExF). This analysis confirmed the results obtained in a previous study [14]. The FTIR analysis presented in this paper shows the presence of hydroxyl groups (-OH) on the surface of the flakes, derived from the native aqueous solution in which the flake suspensions were prepared. Further, the presence of C=O connections may be associated with a greater affinity of the material for the attachment of hydroxyl (OH-) groups. Thus, the more C=O bonds that are hydrophilic in nature, the more OH- hydroxyl groups. The presence of carbonyl groups (C=O) and carboxyl groups (COO-) was also observed. The highest expression was observed in the rGO/TUD treatment. In a study by Loryuenyong et al., the presence of hydroxyl (-OH), carbonyl (C=O), and epoxy (C-O) groups was confirmed in GO and rGO. Moreover, the high intensity of the main peaks in GO confirms the presence of a large number of oxygen functional groups after the oxidation process [25]. Emiru and Ayele obtained a similar band arrangement of the FTIR spectrum of GO and rGO, as presented in this article. Among other things, they observed such functional groups as OH-, COH-, COOH-, and CO- [26]. It showed that with the use of chemical methods, the content of functional groups is reduced. The most effective method for GO reduction is the thermal process because the obtained rGO/Term had the highest rate of reduction [14].

It is known that graphene materials can modulate electron transfer in redox reactions. The speed of the redox reactions on the graphene surface depends on the migration of electrons across the reactive surface and the subsequent transfer along the surface. Pan et al., during an analysis of the resistance in the studied graphene materials, proved that the higher the degree of graphene folding, the more difficult it was to transfer electrons [27]. The same relationship between the degree of folding and resistance was observed in a previous study [14]. Thus, the more corrugated the surface of the carbon material (rGO/Term < rGO/ATS < rGO/TUD), the lower the resistance of the graphene flakes.

The obtained selectivity of graphene flakes towards cancer cells could be determined by the differences in cell membrane potential (Vm) between cancer and noncancerous cells. Cell surface charge is a key biophysical parameter that depends on the composition of the cytoplasmic membrane and the physiological state of cells. Cone’s theory [28] proposes a general correlation between proliferation and Vm, as he showed the significant depolarization of Vm during the malignant transformation of normal cells [29,30]. Direct comparisons of the in vitro and in vivo Vm levels of normal and cancer cells showed that the cancer cells were more depolarized than their normal counterparts [17]. A cell depolarizes when Vm is relatively less negative (tumor cells, proliferating: 0 to −50 mV), while a hyperpolarized cell has more negative Vm (normal cells, nonproliferating; −50 to −90 mV) [31]. Glioblastoma cells express the potential of −14 mV of depolarized cell membrane, which determines the lower repulsive forces. Therefore, the flakes with reduced graphene (rGO/ATS and rGO/TUD in particular) possess a lower negative surface charge and can probably adhere to glioblastoma U87 cell membranes more easily.

When analyzing the results of the cell membrane potential (Vm) of U87 and Hs5, a decrease in cell membrane potential was observed over time after treatment with rGO/ATS and rGO/TUD flakes. However, the decrease in potential in fibroblast Hs5 cells was smaller than that in U87 cells: for rGO/ATS material, it was 258.4 RFU and 421 RFU, respectively, and for rGO/TUD material, 384.3 RFU and 561.7 RFU, respectively. Bondar et al. observed that the membrane potential in HeLa cells, in which apoptosis was thermally induced, shifted negatively by about 4.2 mV compared to control cells. This was probably the result of the redistribution of phosphatidylserine, containing a negatively charged carboxyl group, from the inner to the outer lipid layer of the cell membrane [16]. Therefore, the reduction of the U87 glioma cell membrane potential after treatment with rGO/ATS and rGO/TUD flakes may be partially secondary to the flakes due to the induction of apoptosis in cells.

Graphene flakes, apart from direct contact with the lipid double membrane, are in contact with channels, including ion channels. Ion channels are membrane proteins that open or close a plasmatic membrane, depending on a voltage gradient or ligand binding. They are essential for cell proliferation and play a key role in malignant glioma by influencing the shape and volume of glioblastoma cells, which, in turn, may influence the invasiveness and migration of tumors [32].

The effect of the graphene flakes on the expression of voltage-gated ion channels, participating in both electrical and chemical signaling pathways [33], was assessed. Wang et al. showed that between 18 genes of ion channels (voltage-gated and ligand-gated), the expression of 16 genes (*cacna1d*, *clcn6*, *glrb*, *gria2*, *grid1*, *kcnab1*, *kcnb1*, *kcnd2*, *kcnj10*, *kcnma1*, *kcnqn3*, *nalcn*, *p2rx7*, *scn1a*, and *vdac2*) was reduced compared to normal tissue [34]. Based on these studies, genes encoding voltage-dependent ion channel subunits (*cacna1d*, *clcn6*, *kcnab1*, *kcnb1*, *kcnj10*, and *nalcn*) were selected to determine the effect of graphene derivatives on channel gene expression. The following genes were also added for the analysis: *clcn3* [35], *cacna1b* [36], and *kcne4* [37].

The presented study shows that the expression of *nalcn* in U87 glioma tumor cells was significantly higher (log2RQ = 6.15) than in Hs5 fibroblasts. Ouwerkerk et al. reported that the concentration of Na^+^ ions in malignant tumors increased in comparison to noncancer tissues [38]. Moreover, the expression of *nalcn* in astrocytes, the glial cells that glioblastoma is derived from, was markedly low [39]. The expression of *nalcn* in U87 glioma cells was significantly higher after treatment with rGO/ATS and rGO/TUD. In Hs5 cells, the same effect was observed in the case of each treatment with graphene derivative material. It was suggested that the increase of intracellular Na^+^ occurs in the early phase of apoptosis [40,41]. Moreover, several studies also reported an elevation of cytoplasmic Na^+^ in the late phase of apoptosis [42,43]. Thus, an increased expression of *nalcn*, induced by the graphene flakes, can provoke apoptosis in glioblastoma cells by stimulating sodium influx into the cells, which increases the cytoplasmic concentration of Na^+^.

*clcn6* is predominantly localized in the intracellular vesicles of the endoplasmic reticulum (late endosomes) and the cell membrane. Our study showed a significant increase of *clcn6* during the initial treatment of U87 glioblastoma cells in all tested groups. On the contrary, Hs5 cells revealed a significant increase of *clcn6* expression in the GN/ExF and rGO/ATS treatment groups. The observed overexpression leads to lysosomal acidification [44]. Neagoe et al. clearly proved that CIC-6 mediates the Cl^−^/H^+^ exchange, which affects its coupling, conductivity, and ion selectivity features [45]. Increased expression of the *clcn6* gene in U87 glioma cells leads to cytoplasmic alkalization. In fact, intracellular alkalization and extracellular acidification are commonly observed in malignant tumors. The altered activity of cell transporters, internal enzymes, and pH gradient in the cancer cell membrane plays a pivotal role in tumor progression and metastasis [46,47]. Acidification of the cytoplasm leads to the activation of apoptotic pathways in cancer cells [48]. Based on that, we conclude that graphene flakes (GN/ExF) and rGOs in U87 glioblastoma cells block Cl^−^/H^+^ transport. Consequently, it leads to the acidification of the cytoplasm and the activation of apoptosis. Simultaneously, in response to that blockage, an increase in *clcn6* gene expression can occur.

Four classes of potassium channels are distinguished: Kv channels (voltage-gated), KCa^2+^ channels (calcium-activated), Kir channels (inward-rectifier potassium channel), and K2P channels (two-pore channels) [49,50]. Based on these studies, selected markers of Kv and Kir classes were verified. In the channels of the Kv class, we analyzed expression *kcnb1* (Kv2.1) and *kcne4* channels, as well as *kcnj10*, which belongs to the Kir potassium channel class. Major changes were observed in the expression of the *kcne4* channel belonging to the Kv potassium channel class. This study confirmed a significant increase in the expression of the *kcne4* channel (log2RQ = 1.192) after 24 h treatment of U87 glioma cells with rGO/ATS. Meanwhile, the reduction in *kcne4* expression (log2RQ = −1.734) was observed after 24 h treatment by rGO/TUD. No changes in KCNE4 expression were noted in Hs5 fibroblasts. KCNE4 expression was characterized by a 2.9-fold increase in glioma compared to the healthy tissues [51]. The *kcne4* genes encode single proteins of the transmembrane domain with an extracellular *N*-terminus and an intracellular *C*-terminus. Therefore, the abovementioned proteins cannot form functional ion channels. They can function as the accessory subunits for various ion channels and regulate their biophysical and pharmacological properties in parallel [52,53,54].

The study confirmed no changes in *cacna1b* expression in U87 cells after treatment with different graphene materials. In Hs5 cells, the expression was decreased in all experimental groups in the initial treatment. The results confirmed that the expression of the *cacna1b* gene in the fibroblasts was significantly higher (log2RQ = 7.52) than in U87 cells. Wang et al. reported, based on brain and breast cancer studies, that *cacna1b* is expressed at a low level in tumor cells. Brain cancers, including glioblastoma, oligodendroglia, anaplastic astrocytoma, diffuse astrocytoma, and glioblastoma, show a significant reduction of *cacna1b* expression compared to the control groups [55].

In our studies, we did not observe the expression of the *cacna1d* gene encoding the subunit of calcium channels in U87 cells. Changes in *cacna1d* expression occurred only under rGO influence in Hs5 cells. At the initial 6 h treatment, an increase of *cacna1d* was observed after rGO/Term, rGO/ATS, and rGO/TUD treatment. However, 24 h of treatment decreased its expression in both rGO/ATS and rGO/TUD groups. The primary increase in *cacna1d* expression can induce a greater influx of Ca^2+^ ions into the cells. Therefore, after 24 h of treatment, we noticed the reduction in *cacna1d* expression, which equalized the earlier influx of Ca^2+^ ions.

Analysis of *clcn3* expression in U87 glioma cells showed an increase in expression after treatment with graphene flakes (GN/ExF) and reduced graphene oxide flakes (rGO/Term, rGO/ATS, rGO/TUD). In particular, significant changes were visible in the rGO/ATS and rGO/TUD groups. Recent studies have shown that *clcn3* is highly expressed in GBM, and it plays a significant role in cell survival, proliferation, and malignancy [56,57]. Sontheimer et al. showed that decreased expression of *clcn3* channels inhibits the migration of glioblastoma cells in vitro and in vivo [35]. An increase in *clcn3* expression can stimulate the invasiveness of U87 glioma cells. No parallel changes were observed in Hs5 fibroblasts after treatment with carbon flakes.

Additionally, using the protein matrix, which allows the determination of the expression of 40 different receptor proteins involved in different signaling pathways (Figure 5), we assessed the expression of selected extracellular receptors. As a result, it was noticed that the expression of the uPar protein was increased in U87 cells compared to Hs5 cells. Raghu et al. confirmed significantly higher uPar protein expression in U87 cells in comparison to normal HMEC cells [58]. Analysis of uPar expression in U87 glioma cells after treatment with graphene flakes (GN/ExF) and reduced graphene oxide (rGO/ATS, rGO/TUD) showed a significant reduction of its expression. It was mainly affected by the treatment with reduced graphene oxides. uPar is responsible for the degradation of extracellular matrix (ECM) components attached to the cell surface [59]. It contains three domains connected to the concave structure, which is the binding site for vitronectin. However, since the binding sites of vitronectin and uPa are distinct, uPar can bind simultaneously to both ligands, regulating proteolysis, adhesion, and cell signaling [60]. The association between uPa and uPar can cause the cleavage of the adjacent uPar molecules. The cleaved uPar does not support plasminogen, which is mediated by the activation of uPa on the cell surface [61]. Thus, the flakes of graphene and reduced graphene oxides act similarly to uPa by activating the cleavage of adjacent uPar receptors. They can also block the attachment sites of uPar and vitronectin and, therefore, inhibit uPar signaling [61,62].

Endoglin (CD105) is the other factor that had a changed expression after treatment with GN and rGO flakes. It is transmembrane homodimeric protein localized in the endothelial cells of blood vessels. It is a component of the transforming growth factor β (TGFβ) receptor signaling pathway. CD105 plays a pivotal role in angiogenesis and vasculogenesis processes, preventing apoptosis in hypoxic endothelial cells [63]. It was observed that CD105 is correlated with cancer prognosis (particularly in pediatric cases), but its role in high-grade gliomas remains unclear [64]. Our study showed a significantly higher endoglin expression in U87 glioma cells than in Hs5 fibroblasts. In other studies, a high expression of CD105 in neoplastic tissue, such as in meningiomas [65] or childhood brain tumors [64], was also confirmed. The presented study showed a decrease in CD105 expression in U87 glioma cells after treatment with GN and rGOs in all treatment groups. Muenzner et al. confirmed that the endoglin carboxy-terminal domain is required to inhibit cell detachment [66]. Therefore, treatment with GN and rGO, resulting in decreased endoglin expression, can stimulate cell adhesion and, consequently, leads to the reduction of the ability of cancer cell migration.

## 4. Materials and Methods

### 4.1. Production and Preparation of GN and rGO

Graphene and reduced graphene oxides were supplied by the Łukasiewicz Research Network—Institute of Microelectronics and Photonics, Warsaw. Direct graphite exfoliation using Capstone (a fluorinated surfactant) was used to obtain graphene flakes, designated as GN/ExF. Flakes of rGO were produced by reducing previously prepared graphene oxide (GO). GO was obtained by graphite oxidation and exfoliation, according to a modified Marcano method. Reduced graphene oxide, designated as rGO/ATS, was created by reducing GO with ammonium thiosulphate for 20 h at 95 °C. The molar ratio of the reducing agent to GO was 3:1, and the reduction was conducted at neutral pH. rGO/Term was created by reducing GO powder in an oven at 1000 °C for 1 h under a nitrogen atmosphere. Reduced rGO/TUD graphene oxide was prepared by reducing GO via exposure to thiourea dioxide at 85 °C for 1.5 h. The molar ratio of the reducing agent to GO was 5:1, and pH was set as 9. rGO/ATS and rGO/TUD were purified through pressure filtration on a membrane, and then dialysis was used. During these steps, a significant number of residue chemicals were removed from materials; however, there were still some sulfur ions [14].

### 4.2. Characterization of GN and rGOs

#### 4.2.1. Fourier Transform IR (FT-IR) Spectrometer Analysis

The samples were processed in pellet form. Each pellet consisted of 0.007 g of graphene and 0.200 g of potassium bromide. Infrared absorption of the coatings in the spectral range of 400–4000 cm^−1^ was performed using a model iS50 Fourier transform IR (FT-IR) spectrometer (Thermo Fisher Scientific, Wilmington, DE, USA). Spectra were recorded with a resolution of 4 cm^−1^ using a high sensitivity MCT-B detector (mercury cadmium telluride). The measurements were performed in the transmitation mode. In each case, data from 120 scans were collected to construct a single spectrum.

#### 4.2.2. Scanning Electron Microscopy of Flakes

The morphology of graphene and reduced graphene oxides was examined using scanning electron microscopy (SEM, Hitachi S-3000 N, Minato-ku, Tokyo, Japan). SEM images were taken in the AEE mode to scan the graphene with the table current.

### 4.3. Cell Cultures

Human glioblastoma U87 MG (ATCC^®^ HTB-14™) and bone marrow stromal Hs5 (ATCC^®^ CRL-11882™) cell lines were obtained from the American Type Culture Collection (Manassas, VA, USA) and maintained in Dulbecco’s modified Eagle’s medium (DMEM), supplemented with 10% fetal bovine serum (Life Technologies, Houston, TX, USA) and 1% antibiotic-antimycotic mixture containing penicillin and streptomycin (Life Technologies, Houston, TX, USA). Cultures were maintained at 37 °C under 5% CO_2_ and 95% humidity in an INCOMED153 (Memmert GmbH & Co. KG, Schwabach, Germany).

### 4.4. Cell Membrane Potential Assay

A Cellular Membrane Potential Assay Kit (ab176764, Abcam, Cambridge, UK) was used to detect changes in membrane potential. Cells were plated in 96-well black plates. Approximately 3 × 10^4^ cells were seeded onto each well. After incubation for 24 h, the culture medium was replaced with medium containing GN and rGO at a final concentration of 25 µg/mL. After 24 h of incubation with graphene and graphene derivatives, the growth medium was removed from each well and replaced with 100 μL of diluted (1:10) assay buffer, with the addition of 1.5 μL MP sensor dye loading. The plate was incubated for 30 min at RT in the dark, and membrane potential change was analyzed using an ELISA reader (Infinite M200, Tecan, Durham, NC, USA) by measuring fluorescence at Ex/Em = 530/570 nm.

### 4.5. Isolation of Total RNA and cDNA Synthesis

For the isolation of total RNA, U87 and Hs5 cells (2 × 10^5^) were cultured on a six-well plate in three independent replicates. A water solution of GN and rGO flakes was added to the cultures at a concentration of 25 µg/mL and incubated two independent times for 6 h and 24 h. Cells were detached from the plates by trypsinization, centrifuged for 5 min at 400× *g*, and washed twice with phosphate-buffered saline (PBS; Thermo Fisher Scientific, Wilmington, DE, USA). The cell pellet was resuspended in freshly prepared lysis buffer from a PureLink™ RNA Mini Kit (Thermo Fisher Scientific, Wilmington, DE, USA) containing 1% 2-mercaptoethanol and vortexed at high speed until the cell pellet was completely dispersed and the cells were lysed. The supernatant was transferred to new tubes, mixed with one volume of 70% ethanol, and then transferred to a spin cartridge. Further steps were performed according to the manufacturer’s protocol. The isolated RNA was measured using a NanoDrop 2000 spectrophotometer (Thermo Fisher Scientific, Wilmington, DE, USA). A cDNA High Capacity Reverse Transcription Kit (Applied Biosystems, Foster City, CA, USA) was used. The procedure was performed with the following cycle conditions: 10 min at 25 °C, 120 min at 37 °C, and 5 min at 4 °C using a 2720 Thermal Cycler (Thermo Fisher Scientific, Wilmington, DE, USA). cDNA concentration was measured on a NanoDrop 2000 spectrophotometer and stored for further analysis at −80 °C.

### 4.6. Gene Expression

The reaction was carried out using 48-well plates and Power SYBR™ Green PCR Master Mix (Thermo Fisher Scientific, Wilmington, DE, USA); 100 ng of cDNA was used for each reaction. The following genes were examined: *clcn3*, *clcn6*, *cacna1b*, *cacna1d*, *nalcn*, *kcnj10*, *kcnb1*, and *kcne4*. Gene-specific primers (Table 2) were purchased from Genomed (Warsaw, Poland), and *rpl13a* was used as the reference gene [67]. The reaction was performed using the Step One™ Real-Time PCR System (Thermo Fisher Scientific, Wilmington, DE, USA). Conditions of the reaction were set as specified by the manufacturer. Each sample was analyzed in duplicate. The ΔΔCt method was used to determine mRNA expression by real-time PCR: ΔΔCT = ΔCT test sample—ΔCT calibrator sample. RQ = 2^−ΔΔCT^.

### 4.7. Human Receptor Antibody Array

For protein analysis, glioma cell line U87 and fibroblast cell line Hs5 were treated with graphene (GN) or reduced graphene oxides (rGOs) at a concentration of 25 µg/mL and incubated for 24 h. The cells were scraped off, centrifuged, and washed twice in PBS. Cells not treated with graphene flakes were used as a control. The cell pellet was resuspended in a diluted lysis buffer containing protease and phosphatase inhibitors (Sigma-Aldrich, St. Louis, MO, USA) according to the manufacturer’s instructions. Frozen metal balls and TissueLyser (Qiagen, Hilden, Germany) were used for homogenization at 50 Hz for 10 min on a shaking frozen cartridge. The samples were then centrifuged (30 min; 14,000× *g*; 4 °C), and the supernatant was collected. Protein concentration was determined using a bicinchoninic acid kit (Sigma-Aldrich, St. Louis, MO, USA). Analysis of receptor cell membranes was performed using an antibody array (ab211065; Abcam, Cambridge, UK). The assay was performed in accordance with the manufacturer’s instructions, using lysates containing 400 μg/mL of total protein per membrane. Membranes were visualized using the Azure Biosystem C400 (Azure, Dublin, CA, USA) [63]. The results shown in Figure S1 were obtained by analysis in ImageJ. Results were normalized and compared to a dot control sample.

### 4.8. Statistical Analysis

The data were analyzed using a two-way analysis of variance with GraphPad Prism 8.4.3 (GraphPad Software Inc., La Jolla, CA, USA). Differences between groups were tested using Bonferroni’s multiple comparison test and Dunnett’s multiple comparisons test. All mean values are presented using standard deviation or standard error. Differences at *p* < 0.05 were considered significant.

## 5. Conclusions

Different types of graphene derivatives may activate separate cell pathways. rGOs can affect cells as the result of contact with the glioblastoma cell membrane via its functional groups, which are presented on the surface of the examined flakes. This is the first study confirming that graphene flakes have a significant effect on the expression of voltage-dependent ion channel (VGIC) genes in U87 glioma cells. The mechanism of the effect of graphene-derived flakes on membrane proteins depends on the number of the specific structure of voltage-dependent ion channels and extracellular receptors. It was also demonstrated that major changes in the expression of voltage-dependent ion channel genes were observed in *clcn3*, *nalcn*, and *kcne4* after treatment with rGO/ATS and rGO/TUD flakes. Moreover, we present that the examined graphene forms or different types of graphene derivatives (GN and rGOs) can affect the expression of extracellular receptors (uPar and endoglin) in U87 cells, significantly reducing their expression. We showed that GN and rGOs decrease uPar expression by acting as inhibitors of neoplastic-promoted proteolysis. Therefore, the mobility of mesenchymal-type cells may be inhibited. Moreover, GN and rGOs decrease the expression of endoglin (CD105) and probably provide an increase in cell adhesion, consequently reducing cancer cell migration. In conclusion, we suggest that the presence of oxygen-containing functional groups of rGO, including their number and types, may be the most important feature of the examined flakes for their future medical application.

## Figures and Tables

**Table 1:** Location of absorption bands exhibited by the functional groups in FTIR spectra obtained for individual GN and rGO samples.

No.	Wavenumber [cm^−1^]	Type of Bond	Sample			
			GN/ExF	rGO/Term	rGO/ATS	rGO/TUD
1	3680	O-H (stretch)	-	-	-	+ (w)
2	3600–3000	O-H (stretch)	+ (m)	+ (w)	+ (m)	+ (s)
3	3280	N-H (stretch)	-	-	+ (m)	+ (m)
4	3000–2800	C-H (stretch)	-	+ (m)	+ (m)	+ (s)
5	1747	C=O (stretch)	-	+ (w)	-	-
6	1711	C=O (stretch)	-	-	+ (m)	+ (s)
7	1640	C=C (stretch)	+ (m)	-	+ (m)	+ (s)
8	1563	COO- (stretch)	+ (w)	-	+ (m)	+ (s)
9	1465	C-H (deformation)	-	+ (w)	+ (w)	+ (m)
10	1400	-OH (in-plane bendig)	+ (w)	+ (w)	+ (w)	+ (s)
11	1174	C-O-C (stretch)	+ (w)	-	+ (m)	+ (s)
12	1124	C-NH_2_/C-N (stretch)	-	-	+ (m)	+ (w)
13	1100	C=S (stretch)	-	-	+ (w)	+ (w)
14	1018	Carbon ring	-	+ (w)	+(m)	+(m)
15	800	CH_2_=CH (deformation)	-	-	+ (w)	+ (m)
16	659–511	carbon ring	+ (w)	+ (m)	+ (m)	+ (m)
17	617	C-S (stretch)/S-O (scissors)	-	-	+ (s)	+ (m)

**Notes**: designations used in the table: w—weak; m—medium; s—strong; “+”—current; “-”—absent. **Abbreviations**: GN, graphene; rGO, reduced graphene oxide; ExF, exfoliation; Term, thermal; ATS, ammonium thiosulphate; TUD, thiourea dioxide.

**Table 2:** Primers used to assess the expression of genes involved in voltage-dependant ion channels.

Genes	Forward Primers (5′-3′)	Reverse Primers (5′-3′)	References
*clcn3*	CTGTGCCGCCTCTAAGCC	ACTGTAGTTCGACTCGCTGAA	Primer blast
*clcn6*	ACCTGGAAGTTTTGGAGACCAT	TGAGTTGGGTGAAGAGTCGC	Primer blast
*cacna1b*	CCCTTGCTGTCAACATCTGGT	GGATGGGTGAGGAGTTGGC	Primer blast
*cacna1d*	ACTCGGGCTATCCAGAAGTAG	CTTGCCCAAAGAAAAGACTGC	Primer blast
*nalcn*	CGCCGTAGACTGTGGTTTTG	AATGACGCTGATGATGGCAC	Primer blast
*kcnj10*	TCAGAAGACGGGCGAAACAA	GCGAGCCTAAGCAAGACTCA	Primer blast
*kcnb1*	CCATTCTGCCATACTATGTCACC	AGCAAGCCCAACTCATTGTAG	[68]
*kcne4*	CACCGCTACCTGAAAACCCT	TTGATCGTGGCAGAGTGAGC	Primer blast
*rpl13a*	CATAGGAAGCTGGGAGCAAG	GCCCTCCAATCAGTCTTCTG	[67]

## Data Availability

The data that support the findings of this study are available from the corresponding author upon reasonable request.

## Supplementary Materials

The following are available online at https://www.mdpi.com/1422-0067/22/2/515/s1.

## References

[1] Wen P.Y., Reardon D.A. (2016). Neuro-oncology in 2015: Progress in glioma diagnosis, classification and treatment. Nat. Rev. Neurol..

[2] Zhang Y., Cruickshanks N., Yuan F., Wang B., Pahuski M., Wulfkuhle J., Gallagher I., Koeppel A.F., Hatef S., Papanicolas C. (2017). Targetable T-type Calcium Channels Drive Glioblastoma. Cancer Res..

[3] Chen J.-H., Jang C., Xiao S., Ishigami M., Fuhrer M.S. (2008). Intrinsic and extrinsic performance limits of graphene devices on SiO_2_. Nat. Nanotechnol..

[4] Choi W., Lahiri I., Seelaboyina R., Kang Y.S. (2010). Synthesis of Graphene and Its Applications: A Review. Crit. Rev. Solid State Mater. Sci..

[5] Geim A.K., Novoselov K.S. (2007). The rise of graphene. Nat. Mater..

[6] Compton O.C., Nguyen S.T. (2010). Graphene Oxide, Highly Reduced Graphene Oxide, and Graphene: Versatile Building Blocks for Carbon-Based Materials. Small.

[7] Acik M., Mattevi C., Gong C., Lee G., Cho K., Chhowalla M., Chabal Y.J. (2010). The Role of Intercalated Water in Multilayered Graphene Oxide. ACS Nano.

[8] Gao W. (2015). The chemistry of graphene oxide. Graphene Oxide: Reduction Recipes, Spectroscopy, and Applications.

[9] Grodzik M., Sawosz E., Wierzbicki M., Orlowski P., Hotowy A., Niemiec T., Szmidt M., Mitura K., Chwalibog A. (2011). Na-noparticles of carbon allotropes inhibit glioblastoma multiforme angiogenesis in ovo. Int. J. Nanomed..

[10] Wierzbicki M., Sawosz E., Grodzik M., Kutwin M., Jaworski S., Chwalibog A. (2013). Comparison of anti-angiogenic properties of pristine carbon nanoparticles. Nanoscale Res. Lett..

[11] Chwalibog A., Jaworski S., Sawosz E., Grodzik M., Winnicka A., Prasek M., Wierzbicki M. (2013). In vitro evaluation of the effects of graphene platelets on glioblastoma multiforme cells. Int. J. Nanomed..

[12] Jaworski S., Sawosz E., Kutwin M., Wierzbicki M., Hinzmann M., Grodzik M., Winnicka A., Lipińska L., Wlodyga K., Chwalibog A. (2015). In vitro and in vivo effects of graphene oxide and reduced graphene oxide on glioblastoma. Int. J. Nanomed..

[13] Jaworski S., Strojny B., Sawosz E., Wierzbicki M., Grodzik M., Kutwin M., Daniluk K., Chwalibog A. (2019). Degradation of Mitochondria and Oxidative Stress as the Main Mechanism of Toxicity of Pristine Graphene on U87 Glioblastoma Cells and Tumors and HS-5 Cells. Int. J. Mol. Sci..

[14] Szczepaniak J., Strojny B., Sawosz E., Jaworski S., Jagiello J., Winkowska M., Szmidt M., Wierzbicki M., Sosnowska M., Balaban J. (2018). Effects of Reduced Graphene Oxides on Apoptosis and Cell Cycle of Glioblastoma Multiforme. Int. J. Mol. Sci..

[15] Martelli C., King A., Simon T., Giamas G. (2020). Graphene-Induced Transdifferentiation of Cancer Stem Cells as a Therapeutic Strategy against Glioblastoma. ACS Biomater. Sci. Eng..

[16] Bondar O.V., Saifullina D.V., Shakhmaeva I.I., Mavlyutova I.I., Abdullin T.I. (2012). Monitoring of the Zeta Potential of Human Cells upon Reduction in Their Viability and Interaction with Polymers. Acta Nat..

[17] Marmo A.A., Morris D.M., Schwalke M.A., Iliev I.G., Rogers S. (1994). Electrical Potential Measurements in Human Breast Cancer and Benign Lesions. Tumor Biol..

[18] Fiorillo M., Verre A.F., Iliut M., Peiris-Pagés M., Ozsvari B., Gandara R., Cappello A.R., Sotgia F., Vijayaraghavan A., Lisanti M.P. (2015). Graphene oxide selectively targets cancer stem cells, across multiple tumor types: Implications for non-toxic cancer treatment, via “differentiation-based nano-therapy”. Oncotarget.

[19] Pollak J., Rai K.G., Funk C.C., Arora S., Lee E., Zhu J., Price N.D., Paddison P.J., Ramirez J.-M., Rostomily R.C. (2017). Ion channel expression patterns in glioblastoma stem cells with functional and therapeutic implications for malignancy. PLoS ONE.

[20] Simon O.J., Müntefering T., Grauer O., Meuth S.G. (2015). The role of ion channels in malignant brain tumors. J. Neuro-Oncol..

[21] Parsons D.W., Jones S., Zhang X., Lin J.C.-H., Leary R.J., Angenendt P., Mankoo P., Carter H., Siu I.-M., Gallia G.L. (2008). An Integrated Genomic Analysis of Human Glioblastoma Multiforme. Science.

[22] Joshi A.D., Parsons D.W., Velculescu V.E., Riggins G.J. (2011). Sodium ion channel mutations in glioblastoma patients correlate with shorter survival. Mol. Cancer.

[23] Cuddapah V.A., Robel S., Watkins S., Sontheimer H. (2014). A neurocentric perspective on glioma invasion. Nat. Rev. Neurosci..

[24] Weaver A.K., Liu X., Sontheimer H. (2004). Role for calcium-activated potassium channels (BK) in growth control of human malignant glioma cells. J. Neurosci. Res..

[25] Loryuenyong V., Totepvimarn K., Eimburanapravat P., Boonchompoo W., Buasri A. (2013). Preparation and Characterization of Reduced Graphene Oxide Sheets via Water-Based Exfoliation and Reduction Methods. Adv. Mater. Sci. Eng..

[26] Emiru T.F., Ayele D.W. (2017). Controlled synthesis, characterization and reduction of graphene oxide: A convenient method for large scale production. Egypt. J. Basic Appl. Sci..

[27] Pan M., Zhang Y., Shan C., Zhang X., Gao G., Pan B. (2017). Flat Graphene-Enhanced Electron Transfer Involved in Redox Reactions. Environ. Sci. Technol..

[28] Cone C.D. (1971). Unified theory on the basic mechanism of normal mitotic control and oncogenesis. J. Theor. Biol..

[29] Tokuoka S., Morioka H. (1957). The membrane potential of the human cancer and related cells. I. Gan.

[30] Johnstone B.M. (1959). Micro-Electrode Penetration of Ascites Tumour Cells. Nature.

[31] Yang M., Brackenbury W.J. (2013). Membrane potential and cancer progression. Front. Physiol..

[32] Molenaar R.J. (2011). Ion Channels in Glioblastoma. ISRN Neurol..

[33] Hille B. (2001). Ion Channels of Excitable Membranes.

[34] Wang R., Gurguis C.I., Gu W., Ko E.A., Lim I., Bang H., Zhou T., Ko J.-H. (2015). Ion channel gene expression predicts survival in glioma patients. Sci. Rep..

[35] Sontheimer H. (2008). An Unexpected Role for Ion Channels in Brain Tumor Metastasis. Exp. Biol. Med..

[36] Phan N.N., Wang C.-Y., Chen C.-F., Sun Z., Lai M.-D., Lin Y.-C. (2017). Voltage-gated calcium channels: Novel targets for cancer therapy. Oncol. Lett..

[37] Solé L., Roura-Ferrer M., Pérez-Verdaguer M., Oliveras A., Calvo M., Fernández-Fernández J.M., Felipe A. (2009). KCNE4 suppresses Kv1.3 currents by modulating trafficking, surface expression and channel gating. J. Cell Sci..

[38] Ouwerkerk R., Jacobs M.A., Macura K.J., Wolff A.C., Stearns V., Mezban S.D., Khouri N.F., Bluemke D.A., Bottomley P.A. (2007). Elevated tissue sodium concentration in malignant breast lesions detected with non-invasive 23Na MRI. Breast Cancer Res. Treat..

[39] Cahoy J.D., Emery B., Kaushal A., Foo L.C., Zamanian J.L., Christopherson K.S., Xing Y., Lubischer J.L., Krieg P.A., Krupenko S.A. (2008). A Transcriptome Database for Astrocytes, Neurons, and Oligodendrocytes: A New Resource for Understanding Brain Development and Function. J. Neurosci..

[40] Fernández-Segura E., Cañizares F.J., Cubero M.A., Warley A., Campos A. (1999). Changes in Elemental Content During Apoptotic Cell Death Studied by Electron Probe X-Ray Microanalysis. Exp. Cell Res..

[41] Skepper J.N., Karydis I., Garnett M.R., Hegyi L., Hardwick S.J., Warley A., Mitchinson M.J., Cary N.R.B. (1999). Changes in elemental concentrations are associated with early stages of apoptosis in human monocyte-macrophages exposed to oxidized low-density lipoprotein: An X-ray microanalytical study. J. Pathol..

[42] Arrebola F., Fernández-Segura E., Campos A., Crespo P.V., Skepper J.N., Warley A. (2006). Changes in intracellular electrolyte concentrations during apoptosis induced by UV irradiation of human myeloblastic cells. Am. J. Physiol. Cell Physiol..

[43] Arrebola F., Zabiti S., Cañizares F.J., Cubero M.A., Crespo P.V., Fernández-Segura E. (2005). Changes in intracellular sodium, chlorine, and potassium concentrations in staurosporine-induced apoptosis. J. Cell. Physiol..

[44] Poët M., Kornak U., Schweizer M., Zdebik A.A., Scheel O., Hoelter S., Wurst W., Schmitt A., Fuhrmann J.C., Planells-Cases R. (2006). Lysosomal storage disease upon disruption of the neuronal chloride transport protein ClC-6. Proc. Natl. Acad. Sci. USA.

[45] Neagoe I., Stauber T., Fidzinski P., Bergsdorf E.-Y., Jentsch T.J. (2010). The Late Endosomal ClC-6 Mediates Proton/Chloride Countertransport in Heterologous Plasma Membrane Expression. J. Biol. Chem..

[46] Webb B.A., Chimenti M.S., Jacobson M.P., Barber D.L. (2011). Dysregulated pH: A perfect storm for cancer progression. Nat. Rev. Cancer.

[47] Damaghi M., Wojtkowiak J.W., Gillies R.J. (2013). pH sensing and regulation in cancer. Front. Physiol..

[48] Matsuyama S., Reed J.C. (2000). Mitochondria-dependent apoptosis and cellular pH regulation. Cell Death Differ..

[49] Liu J., Qu C., Han C., Chen M.-M., An L.-J., Zou W. (2019). Potassium channels and their role in glioma: A mini review. Mol. Membr. Biol..

[50] Du J., Haak L.L., Phillips-Tansey E., Russell J.T., McBain C.J. (2000). Frequency-dependent regulation of rat hippocampal somato-dendritic excitability by the K^+^ channel subunit Kv2.1. J. Physiol..

[51] Biasiotta A., D’Arcangelo D., Passarelli F., Nicodemi E.M., Facchiano A. (2016). Ion channels expression and function are strongly modified in solid tumors and vascular malformations. J. Transl. Med..

[52] McCrossan Z.A., Abbott G.W. (2004). The MinK-related peptides. Neuropharmacology.

[53] Li Y., Sung Y.U., McDonald T.V. (2006). Voltage-Gated Potassium Channels: Regulation by Accessory Subunits. Neuroscientist.

[54] Kanda V.A., Abbott G.W. (2012). KCNE Regulation of K^+^ Channel Trafficking—A Sisyphean Task?. Front. Physiol..

[55] Wang C.-Y., Lai M.-D., Phan N.N., Sun Z., Lin Y.-C. (2015). Meta-Analysis of Public Microarray Datasets Reveals Voltage-Gated Calcium Gene Signatures in Clinical Cancer Patients. PLoS ONE.

[56] Hong S., Bi M., Wang L., Kang Z., Ling L., Zhao C. (2015). CLC-3 channels in cancer (Review). Oncol. Rep..

[57] Lui V.C.H., Lung S.S.S., Pu J.K.S., Hung K.N., Leung G.K.K. (2010). Invasion of human glioma cells is regulated by multiple chlo-ride channels including ClC-3. Anticancer Res..

[58] Montuori N., Cosimato V., Rinaldi L., Rea V.E.A., Alfano D., Ragno P. (2013). uPAR regulates pericellular proteolysis through a mechanism involving integrins and fMLF-receptors. Thromb. Haemost..

[59] Raghu H., Lakka S.S., Gondi C.S., Mohanam S., Dinh D.H., Gujrati M., Rao J.S. (2010). Suppression of uPA and uPAR Attenuates Angiogenin Mediated Angiogenesis in Endothelial and Glioblastoma Cell Lines. PLoS ONE.

[60] Huai Q., Zhou A., Lin L., Mazar A.P., Parry G.C., Callahan J., Shaw D.E., Furie B., Furie B.C., Huang M. (2008). Crystal structures of two human vitronectin, urokinase and urokinase receptor complexes. Nat. Struct. Mol. Biol..

[61] Høyer-Hansen G., Rønne E., Solberg H., Behrendt N., Ploug M., Lund L.R., Ellis V., Danø K. (1992). Urokinase plasminogen activator cleaves its cell surface receptor releasing the ligand-binding domain. J. Biol. Chem..

[62] Høyer-Hansen G., Behrendt N., Ploug M., Danø K., Preissner K.T. (1997). The intact urokinase receptor is required for efficient vitronectin binding: Receptor cleavage prevents ligand interaction. FEBS Lett..

[63] Smith S.J., Tilly H., Ward J.H., MacArthur D.C., Lowe J., Coyle B., Grundy R.G. (2012). CD105 (Endoglin) exerts prognostic effects via its role in the microvascular niche of paediatric high grade glioma. Acta Neuropathol..

[64] Birlik B., Canda S., Ozer E. (2006). Tumour vascularity is of prognostic significance in adult, but not paediatric astrocytomas. Neuropathol. Appl. Neurobiol..

[65] Barresi V., Cerasoli S., Vitarelli E., Tuccari G. (2007). Density of microvessels positive for CD105 (endoglin) is related to prognosis in meningiomas. Acta Neuropathol..

[66] Muenzner P., Rohde M., Kneitz S., Hauck C.R. (2005). CEACAM engagement by human pathogens enhances cell adhesion and counteracts bacteria-induced detachment of epithelial cells. J. Cell Biol..

[67] Aithal M.G.S., Rajeswari N. (2015). Validation of Housekeeping Genes for Gene Expression Analysis in Glioblastoma Using Quantitative Real-Time Polymerase Chain Reaction. Brain Tumor Res. Treat..

[68] Wang H.-Y., Wang W., Liu Y.-W., Li M.-Y., Liang T.-Y., Li J.-Y., Hu H.-M., Lu Y., Yao C., Ye Y.-Y. (2017). Role of KCNB1 in the prognosis of gliomas and autophagy modulation. Sci. Rep..

