# Supplementary material for: Reduced Graphene Oxides Modulate the Expression of Cell Receptors and Voltage-Dependent Ion Channel Genes of Glioblastoma Multiforme
Source: Int J Mol Sci. 2021 Jan 6;22(2):515. doi: 10.3390/ijms22020515 (PMC7825604; doi:10.3390/ijms22020515)
Supplement: Supplementary file 1 [file ijms-22-00515-s001.pdf]

Supplementary Materials:

Table S1 Changes in the expression of voltage-dependant ion channels in U87 (glioblastoma multiforme cell line) and Hs5 (fibroblast cell line). **Notes:** n.e. means non-expression.

**Cell line**		**U87**				**Hs5**			
**Treatment**		**Classification**	**Gene**	**RQ**	**Log$_2$RQ**	**Classification**	**Gene**	**RQ**	**Log$_2$RQ**
**GN/ExF**	**6 h**	**Upregulated**	*CLCN6*	1,3331979	0,400900841	**Upregulated**	*CLCN6*	1,016570355	0,021419525
			KCNE4	1,122563279	0,146626949		*NALCN*	2,958499955	0,841217359
			CLCN3	1,89793895	0,742386182		*CACNA1D*	1,372780285	0,424427986
			CACNA1B	2,744087912	1,204350662		*KCNJ10*	2,061654764	0,952134768
			NALCN	1,125816509	0,159136772		*KCNB1*	1,023783232	0,028834343
			KCNJ10	1,288103191	0,160208066	**Downregulated**	*CLCN3*	0,838584717	-0,358846982
			KCNB1	1,177100265	0,034480572		*CACNA1B*	0,687431891	-0,968313217
		**No expression**	*CACNA1D*	n.e.	n.e.		*KCNE4*	1,020293525	-0,011341095
	**24 h**	**Upregulated**	*CLCN6*	1,092756053	0,113399188	**Upregulated**	*CLCN6*	1,192931597	0,204937935
			CLCN3	1,663099571	0,63972187		*CLCN3*	1,187820858	0,122150421
			CACNA1B	2,401439607	0,615292549		*CACNA1B*	1,22040526	0,21636041
			NALCN	1,566559326	0,644545237		*NALCN*	18,69398059	3,323841095
			KCNB1	1,583914535	0,643200876		*KCNJ10*	2,482231606	0,96110789
		**Downregulated**	*KCNE4*	0,657972875	-0,619467735	**Downregulated**	*KCNE4*	0,840278314	-0,477552414
			KCNJ10	0,556881529	-1,047450701		*KCNB1*	0,922932049	-0,335605621
		**No expression**	*CACNA1D*	n.e.	n.e.		*CACNA1D*	0,908556443	-0,220032374
**rGO/Term**	**6 h**	**Upregulated**	*CLCN6*	1,300059571	0,367934863	**Upregulated**	*CACNA1D*	1,849712742	0,77661864
			CLCN3	2,004422686	0,932834307		*NALCN*	1,659462977	0,253767014
			CACNA1B	10,81710268	1,4016325		*KCNJ10*	1,344118864	0,275710742
			NALCN	1,112613076	0,124638875	**Downregulated**	*CLCN6*	0,932530706	-0,106302579
			KCNB1	1,219914067	0,244347572		*CLCN3*	0,554210394	-0,890827497
			KCNE4	1,341633907	0,368197441		*CACNA1B*	0,131362976	-2,946339607
		**Downregulated**	*KCNJ10*	1,144124513	-0,133036613		*KCNB1*	0,908833993	-0,14484024
		**No expression**	*CACNA1D*	n.e.	n.e.		*KCNE4*	1,037002155	-0,077622414
	**24 h**	**Upregulated**	*CLCN6*	1,003580365	0,000738462	**Upregulated**	*CLCN3*	1,2777634	0,30
			CLCN3	1,217675556	0,25805982		*CACNA1B*	1,018777877	0,004713376
			NALCN	1,25114783	0,297418276		*NALCN*	10,75699264	3,196584702
		**Downregulated**	*CACNA1B*	0,351831389	-1,8005627		*KCNE4*	1,354328026	0,356863022
			KCNJ10	0,578512823	-1,167643865	**Downregulated**	*CLCN6*	0,840714247	-0,26
			KCNB1	1,009151031	-0,141378403		*CACNA1D*	0,888942164	-0,343770345
			KCNE4	0,835495639	-0,355083466		*KCNJ10*	1,010483985	-0,265478134
		**No expression**	*CACNA1D*	n.e.	n.e.		*KCNB1*	0,909271447	-0,20776844
**rGO/ATS**	**6 h**	**Upregulated**	*CLCN6*	1,343214992	0,424389521	**Upregulated**	*CLCN6*	1,396207767	0,47530365
			CLCN3	1,329855543	0,294888814		*CACNA1D*	2,667572338	1,140621503
			CACNA1B	6,055479992	1,984277725		*NALCN*	2,999447093	1,008925438
			KCNJ10	2,286408576	0,877258937		*KCNJ10*	2,376563053	1,088429451
			KCNE4	1,773190548	0,820899487		*KCNE4*	1,118353742	0,068469524
		**Downregulated**	*NALCN*	0,988821753	-0,02704366	**Downregulated**	*CLCN3*	0,716009954	-0,533124606
			KCNB1	0,54167406	-1,261337757		*CACNA1B*	0,22850311	-2,138197581
		**No expression**	*CACNA1D*	n.e.	n.e.		*KCNB1*	0,663146579	-0,598874569
	**24 h**	**Upregulated**	*CLCN6*	1,064915719	0,071888288	**Upregulated**	*CLCN3*	1,097868277	0,093849818
			CLCN3	79,92947918	4,671477		*CACNA1B*	1,19287179	0,220554988
			NALCN	6,835126445	2,468915304		*NALCN*	9,258582354	2,418137868
			KCNJ10	1,82768721	0,711835225		*KCNJ10*	1,819976294	0,77433904
			KCNB1	2,430575382	1,204109192		*KCNE4*	1,104428115	0,122511864
			KCNE4	2,396475045	1,242078781	**Downregulated**	*CLCN6*	0,902238748	-0,150525729
		**Downregulated**	*CACNA1B*	0,481708549	-1,866898219		*CACNA1D*	0,605564771	-0,785147985
		**No expression**	*CACNA1D*	n.e.	n.e.		*KCNB1*	0,612232986	-0,924949646
**rGO/TUD**	**6 h**	**Upregulated**	*CLCN6*	1,314329253	0,386397362	**Upregulated**	*CLCN6*	1,206013254	0,264055888
			CLCN3	2,312135547	1,137585322		*CACNA1D*	3,293277232	1,367697398
			NALCN	1,088084495	0,087058385		*KCNJ10*	2,303738053	0,961934408
			KCNJ10	1,313874157	0,25367101	**Downregulated**	*CLCN3*	0,852644485	-0,252981186
			KCNB1	1,158837812	0,162621498		*CACNA1B*	0,208935779	-2,276677767
		**Downregulated**	*CACNA1B*	8,889092861	-1,275837072		*NALCN*	0,817521193	-0,825876236
			KCNE4	0,982379429	-0,030621529		*KCNB1*	0,949172443	-0,113222599
		**No expression**	*CACNA1D*	n.e.	n.e.		*KCNE4*	0,749059004	-0,516274929
	**24 h**	**Upregulated**	*CLCN3*	128,8257586	6,894676844	**Upregulated**	*CLCN3*	1,199336796	0,212770144
			NALCN	9,177492075	3,196760178		*NALCN*	8,875269206	2,851837794
			KCNJ10	2,734078391	1,210272471		*KCNJ10*	1,589295573	0,451617241
			KCNB1	1,336270569	0,236512661	**Downregulated**	*CLCN6*	0,85243567	-0,230883916
		**Downregulated**	*CLCN6*	0,881006191	-0,186611176		*CACNA1B*	0,999821046	-0,032211304
			CACNA1B	0,402066592	-1,636833827		*CACNA1D*	0,605288154	-1,046854973
			KCNE4	0,386190656	-1,684004307		*KCNB1*	0,822369415	-0,357221127
		**No expression**	*CACNA1D*	n.e.	n.e.		*KCNE4*	0,918612557	-0,271642208

Table S2 Full array map of analysis membrane receptors in U87 and Hs5 cells.

	A	B	C	D	E	F	G	H	I	J	K	L
**1**	POS	POS	NEG	NEG	4-1BB	ALCAM	CD80	BCAM	CD14	CD30	CD40 Ligand	CEACAM-1
**2**	POS	POS	NEG	NEG	4-1BB	ALCAM	CD80	BCMA	CD14	CD30	CD40 Ligand	CEACAM-1
**3**	Dr6	Dtk	Endoglin	ErbB3	E-Selectin	Fas	Flt-3	GITR	HVEM	ICAM-3	IL-1 R4	IL-1 R1
**4**	Dr6	Dtk	Endoglin	ErbB3	E-Selectin	Fas	Flt-3	GITR	HVEM	ICAM-3	IL-1 R4	IL-1 R1
**5**	IL-10R beta	IL-17RA	IL-1R gamma	IL-21R	LIMPII	Lipocalin-2	L-Selectin	LYVE-1	MICA	MICB	NRG-1 beta	PDGF R beta
**6**	IL-10R beta	IL-17RA	IL-1R gamma	IL-21R	LIMPII	Lipocalin-2	L-Selectin	LYVE-1	MICA	MICB	NRG-1 beta	PDGF R beta
**7**	PEACAM-1	RAGE	TM-1	TRAIL R3	Trappin-2	uPar	VAM-1	XEDAR	BLANK	BLANK	BLANK	POS
**8**	PEACAM-1	RAGE	TM-1	TRAIL R3	Trappin-2	uPar	VAM-1	XEDAR	BLANK	BLANK	BLANK	POS

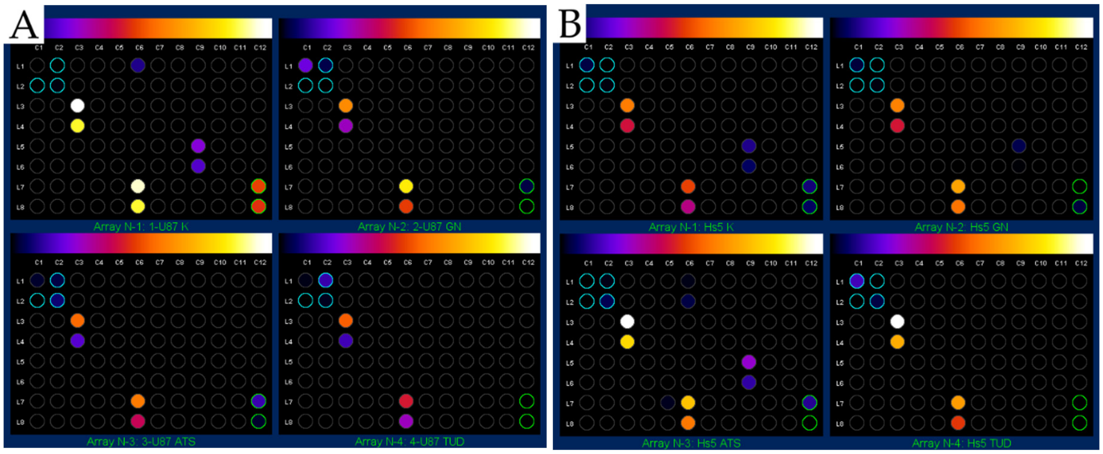

Figure S1 Antibody array analysis of human cell membrane receptor in U87 glioma cells (A) and Hs5 fibroblast cells (B) with or without treatment (K; control) with graphene flakes (GN/ExF) and two types of reduced graphene oxides (rGO/ATS and rGO/TUD). The results shown in the figure were obtained by analysis in ImageJ. The dots with the location (C3-L3, C3-L4) indicate the expression Endoglin (CD105), (C6-L7, C6-L8) indicate uPar (CD87), and (C9-L5, C9-L6) indicate MICA.
